# Directions for Using Dr. A. J. Watts' Improved Crystal Gold

**Published:** 1855-01

**Authors:** 


### ARTICLE X.

### *Directions for using Dr. A. J. Watts' Improved Crystal Gold.*

In using the crystal gold, it will be necessary, in order to secure the highest degree of success, to take into consideration the nature and character of the article to be used, wherein it differs from foil, and the peculiar manner of treatment it requires, for in many respects, the system of manipulations which would secure the best foil stopping, would utterly fail of success when applied to crystal gold.

Crystal gold, as its name indicates, is made up of a combination of crystals of pure gold, so interlaced and interwoven, that upon being submitted to pressure it readily welds into a solid mass. By leaving its upper surface rough in the act of filling, layer upon layer may be built upon itself until any desirable thickness is attained.

As the arrangement of the crystals of the gold furnished, are in the most perfect condition for complete consolidation, it should be the study of the operator, to avoid breaking up the formation any more than is absolutely necessary, and, so far as possible, to give a direct pressure upon the gold—on a line with itself, especially in the early part of the operation. To this end, it will be necessary to be furnished with numerous instruments, whose extremities nearly correspond in breadth to the cavities to be stopped; these *primary* instruments should be so constructed that their diameters for a short distance from their plugging extremities, will correspond with them, in order to avoid any lateral pressure, which would have a tendency to break up or separate the mass into particles. All wedge-shaped instruments should be dispensed with in *introducing* the gold into the cavity, they will come in use after the gold is fixed to its place, and partly condensed. If you wish to have gold carried to lateral points in the cavity, you can do so by taking smaller instruments of the same character, armed with smaller masses of gold and carrying them to their place direct.

After the gold is fixed in its place, and partly consolidated with broad instruments, they may be followed with smaller ones, and then with blades and points, as for foil. Great care should be taken during the progress of filling, to consolidate the gold about the edges of the cavity, so as to secure good and impervious *joints;* pursuing this course until the cavity is full, you then pass over the entire surface with a pointed plugger, and finish in the usual manner.

The instruments referred to, should have the face of their points cut with a file so as to resemble the surface of a fine burr instrument, by this means, the operator is not only enabled readily to take up the gold with the instruments themselves, but also to constantly ensure an irregular and broken surface for the reception of the next layer of gold. These instruments can be modified to any size and shape, or bent to any required angle, providing their points preserve the same general character. For lateral and approximal cavities, thin blades, wedge-shaped, and curved instruments with roughened surfaces, are indispensable for carrying the gold to its place, and partly consolidating it; afterwards they may be followed by the ordinary instruments used for foil, providing their points are serrated.

It is desirable to introduce as large masses as practicable into the cavity, especially in the early part of the operation, for the reason that they can be as readily and completely consolidated as those of less size. The character of this gold so differs from that of foil, that even when introduced in masses, upon direct pressure being applied, it so yields upon itself, that it may be readily carried to the remotest point of the cavity, and there consolidated against its wall. Unlike the smooth surface of foil, it presents myriads of angles to the opposing bone, and insinuates itself into the texture of the tooth; for this reason, and also that the gold becomes constantly, and absolutely solid as the operation advances, it is not necessary as much as heretofore, to *form* cavities to ensure the retention of the gold.

The most successful operators with foil, succeed best when they prepare their gold in the form of *pellets*, which are constructed by breaking up the smooth surface of the foil into as many angles as possible, and shaping it into balls or pellets of different sizes; these are introduced one by one into the cavity of the tooth, where they readily take hold upon those which have preceded them, when they are gradually worked into their texture.

The crystal gold comes to you in a condition vastly superior to any prepared foil in this respect, and in a form from which you may readily cut off pellets of any desirable size or character.

It will be well for those who have not had experience in the use of crystal gold, to confine their operations for a while to that class of cavities which can be directly and easily approached; after they have acquired experience in the material, the tools and their manipulation, lateral and more difficult cavities may be attempted.

In case the gold should become wet during the operation of filling, thoroughly condense the gold already in the cavity, then burnish its surface and let the patient rest.

In resuming the operation, brush out any crumbles of gold that may remain, dry the cavity and surface of gold with coils of tissue or bibulous paper, then, with a sharp pointed condensing instrument, stipple over the entire face of the stopping, taking advantage of all irregularities or undercuts of the surface; again dry the gold with paper and proceed as before the accident, only taking care to work the first succeeding layer of gold thoroughly into the texture of that which it overlies.

In case the gold loses its adhesive quality by dampness or exposure, simply drying it over a spirit lamp will restore it to its original condition; additional heat will increase its density according to your desire.

The numbers indicate difference as to density. No. 1, is soft, tenacious and bulky. No. 2, is more dense and less bulky, the degree of density increasing to No. 4, which is comparatively dense and hard, though still retaining its plastic and working qualities.

